# Functional Traits in Bees: the Role of Body Size and Hairs in the Pollination of a Passiflora Crop

**DOI:** 10.1007/s13744-023-01058-w

**Published:** 2023-07-26

**Authors:** Angela M. Cortés-Gómez, Adrián González-Chaves, Nicolás Urbina-Cardona, Lucas A. Garibaldi

**Affiliations:** 1 Facultad de Estudios Ambientales y Rurales, Pontificia Univ Javeriana, Bogotá, Colombia; 2 Depto de Ecología, Instituto de Biociências, Univ de São Paulo, São Paulo, Brazil; 3 Instituto de Investigaciones en Recursos Naturales, Agroecología y Desarrollo Rural, Univ Nacional de Rio Negro, Bariloche, Río Negro, Argentina; 4 Consejo Nacional de Investigaciones Científicas y Técnicas, Agroecología y Desarrollo Rural, Instituto de Investigaciones en Recursos Naturales, Río Negro, Argentina

**Keywords:** Apidae, Body size, Hairiness, Sweet granadilla, Pollen, Pollination

## Abstract

Pollination is a vitally important function in nature and becomes an ecosystem service because it influences the food and nutritional security for people. However, the contribution of different functional traits of insects for pollen transport of plants is still poorly known. We explore the relationship between pollinator insect functional traits and the transport of pollen of sweet granadilla (*Passiflora ligularis* Juss) in eight crops. We sampled flower-visiting insects of this crop and recorded 10 functional traits (five by direct measurements and five from the literature) that were related to the amount of pollen carried by each insect. Bees (Apidae) were not only the most abundant insects but also the ones that loaded the highest amounts of pollen. Within these, the most abundant species was the exotic common honeybee (*Apis mellifera* (Linnaeus)) making up almost half of the specimens collected; however, this bee carried less pollen grains than other native bees. *Bombus hortulanus* (Smith) was one of the large-bodied native bees that carried more sweet granadilla pollen, despite not being an abundant species in the community. Body size was the most important trait determining the transport of sweet granadilla pollen, while the traits related to body hairs were not significant for the body’s pollen load. None of the functional traits evaluated was influenced by taxonomy at species-level. Our results suggest that large body sizes in bees are the most important traits in granadilla pollen transport, regardless of other changes in composition and structure of pollinating insect assemblages in the crop.

**Supplementary Information:**

The online version contains supplementary material available at 10.1007/s13744-023-01058-w.

## Introduction

Pollination represents a critical ecosystem function in life support that is crucial for planetary ecological stability and the provision of services and resources for food production (Crenna et al. 2017). Likewise, it is of great importance in the maintenance of genetic diversity of wild plant communities in natural ecosystems and in agricultural production (Potts et al. 2010). Approximately 70% of 1330 tropical crop types benefit from animal pollination (Roubik and (Ed.) 1995). Furthermore, pollinators can increase the production of approximately 75% of the world’s 115 most important crop types, measured by food production and economic value (Klein et al. 2007; Gallai et al. 2009; Garibaldi et al. 2011).

There is a growing consensus that the diversity and variability of functional traits, called trait-based ecology, is one of the most relevant components of biodiversity for understanding the functioning of ecosystems (Shipley et al. 2016). Changes in the value of functional traits help us to understand in a more consistent way crop yields (Woodcock et al. 2019) or species responses to environmental changes, than does taxonomic diversity (Moretti et al. 2009; Shipley et al. 2016). This approach tries to establish causal relationships between functional traits (physiological, morphological, or behavioral) and ecosystem processes, functions, and services that are key roles in maintaining the planet’s life support ecosystems (Martín-López et al. 2007).

A wide variety of morphological and behavioral characteristics that contribute to pollination functions have been identified, both in plants and in the animals that pollinate them (Mayfield et al. 2006; Munyuli 2014; Fornoff et al. 2017); these are typically referred to as effect traits (Woodcock et al. 2019). Some of the proposed traits that mediate pollination by insects are sociality, flight range, nesting, body size, dietary specialization, and hairiness (da Encarnacao Coutinho et al. 2018; Borges et al. 2020). The latter one has been often studied recently, thanks to new techniques developed for its measurement (Khan and Liu 2022; Goulnik et al. 2020).

However, despite the increase in effect traits research, little is known about which and how these functional traits contribute to pollination function, specifically in one of the processes of acquisition of pollen by pollinators that is one of the first steps of pollen transport and transfer (Woodcock et al. 2019; Cullen et al. 2021). For this reason, we aim to assess the functional traits of an insect community that better predicts efficiency (amount) in the transport of the pollen of sweet granadilla (*Passiflora ligularis* Juss) in crops. The sweet granadilla belongs to the Passifloraceae family that is a fruit native to South America. It is a vine with a herbaceous stem, woody towards the base. The flowers are violet, showy and have a pleasant aroma that measure between 7 and 10 cm in diameter. Its flowers are cross-pollinated hermaphrodites (Ocampo Pérez 2007; Melgarejo 2015). Worldwide, Colombia is the main producer of sweet granadilla with 4500 cultivated ha and an approximate production of 55,000 t/year. This crop is very important for the income of small farmers, who cultivate on average areas that do not exceed 1.5 hectares (Parra 2013).

Therefore, this work aims to determine which are the functional traits of insects visiting sweet granadilla crops that are involved in pollen transport by assessing the following research questions: Which are the main flower-visiting insects that transport pollen of various plant species and specific pollen of sweet granadilla? How are the functional traits related to each other and how do they associate with the species? What functional traits are most important in the transport of granadilla pollen?

## Materials and methods

### Study area

We conducted this study in Algeciras municipality, department of Huila, on the south-eastern slope of the Andean Cordillera in Colombia, between 800 and 3000 m above sea level with an average minimum temperature of 18 °C and a maximum of 26 °C and two rainy seasons (January–May and October–December) and one dry season (June–September). Most land in the municipality has steep slopes (50% or higher) and the life zones correspond to a transition between cloud-submontane forest and tropical dry forest; 22% of the total municipal land is dedicated to the cultivation of crops such as coffee, sugarcane, banana, sweet granadilla, and passion fruit (PDT-Algeciras 2016-2019). We selected eight farms that produced sweet granadilla crops with an average field size of 1 ha and that were in reproductive state. Farms were separated by a minimum of 3 km between them, and they had similar altitudes and were within the same life zone of cloud-submontane forest.

### Sampling of species and pollen collection

Two persons randomly walked each crop per farm searching for insects. Insect sampling lasted 30 min per site and was repeated each hour, between 6:00 and 16:30 h. Biological material was collected during 2 days per farm during March–April and July–August in 2018 to assess insect communities during the rainy and the dry season. We captured insects that had contact with granadilla flower reproductive structures using a small glass jar containing a rapid killing agent (ethyl acetate). These captured insects were considered as potential pollinators. We identified these insects to species. In case of misidentification, specimens were assigned to a morphospecies. Specimens were deposited in the bee scientific collection (Laboratorio de investigación en Abejas Nativas — LABUN) of the Universidad Nacional de Colombia.

We dabbed the back (thorax) and abdomen of individuals with a 2-mm cube of glycerinated jelly (Howlett et al. 2011) to collect body pollen (free pollen grains carried by insects, Parker et al. 2015) samples. We avoided touching the corbicula or scopa structures. These samples were stored in 1.5-ml sterile vials, transported, and preserved in the LABUN of the Universidad Nacional de Colombia. Only optimal samples (with readable labels or no dirt on the sample) were analyzed. The cube of jelly was placed on a clean microscope slide, melted, and then covered with a microscope cover slip. This slide was examined under an optical microscope coupled to the image capture system (Leica ICC50 HD, Wetzlar, Germany) mainly with the 40 × objective, occasionally changing to 100 × to take photos. The photos were taken with Leica LAS EZ 3.4 photo processing software. We counted all the pollen grains per sample, regardless of being sweet granadilla pollen or not, (from here called, “total pollen”). We also identified and counted separately the sweet granadilla pollen (from here, “granadilla pollen,” see Fig. S1, Supplementary Materials).

Only the pollen data belonging to species that had more than three individuals in the community were analyzed. However, even though the *Diasiops* sp. (Rondani, Diptera) has more than three individuals, it was excluded from the analysis, since it is a pest species for cultivation that damages flowers, and the body size of this insect is too small to take the pollen sample (< 1 mm; Hernández et al. 2011).

### Functional traits of insects

For all insects collected, we measured ten continuous individuals (body size, length left wing, wingspan, length hair length, amount of body hair) and categorical (structures to carry pollen, social behavior, nest location, feeding habit) functional traits. The data of categorical traits were obtained from the literature (see Table S1). Six of these traits (body size, social behavior, nest location, structures to carry pollen, and feeding habit) have been widely used in the literature to evaluate pollination (Woodcock et al. 2019; see Table S1).

In order to measure body size, hair length, and wingspan, we photographed the dorsal and lateral view of each one with a digital camera adapted to a Leica M165C stereo microscope (Dino‐Lite Edge; AnMo Electronics Corporation). Measurements were carried out using the software ImageJ (Version 1.52a, Schneider et al. 2012). For hair length, we measured three hairs in different sites on the dorsal part of the insect, and then we calculated a mean value for each individual. This is because some specimens showed different sizes of hair on the thorax, also to have a greater robustness in the data (see Fig. S2). 

### Data analysis

We analyzed the relationship between functional traits and the species using a cluster analysis for mixed types to combine categorical traits (e.g., nesting site) and continuous traits (e.g., body size). We calculated the Gower distance matrix for selected traits and then did a cluster analysis through the algorithm PAM (partitioning around medoids). We selected the number of clusters with the function “silhouette width”; this metric can range from − 1 to 1, where higher values are better to select (Kaufman and Rousseeuw 2005). After selecting the number of clusters, we identified which species was assigned to which specific cluster.

To test which functional traits were associated with the highest amounts of granadilla and total pollen, we used generalized linear mixed-effects models with functional traits as fixed effects as variables and insect species and granadilla crops as a random effects. The model coefficients were estimated using the package lme4 (Bates et al. 2015) in R free software (R Development Core Team 2020). We considered a negative binomial distribution of the pollen abundance variable to avoid overdispersion issues and as a discrete variable. We created a full model using all the non-correlated functional trait variables by previously excluding variables with a Spearman correlation higher than 0.7 using the corrplot function on the tydyverse package (Wickham et al. 2019). Moreover, we verified that there were no multicollinearity issues in the full model using the vif function from the car package (Fox and Weisberg 2019). We proceeded to select the best fixed structure and calculated the relative importance of the fixed variables using the dredge function and Akaike information criterion (AICc) with the MuMIn package (Bartón 2020). To calculate the relative importance for each variable, we used the importance function from the MuMIn package (Bartón 2020) that considers the Akaike weights of each model that contains the parameter of interest.

## Results

### Visitor species of sweet granadilla flowers

In total, 612 individuals visited the sweet granadilla flowers belonging to 19 insect families; most of the individuals (56.2%) were *Apis mellifera* (Linnaeus). Pollen analysis was performed on 214 individuals of 29 species that met the sample selection criteria. The individuals belonged to two orders: Diptera with four (4) families and five (5) species, and Hymenoptera with five (5) families and 24 species. The Apidae had the largest number of species (Table S2). A total of 33,512 pollen grains were counted of which 3912 were sweet granadilla pollen.

**Fig. 1:**
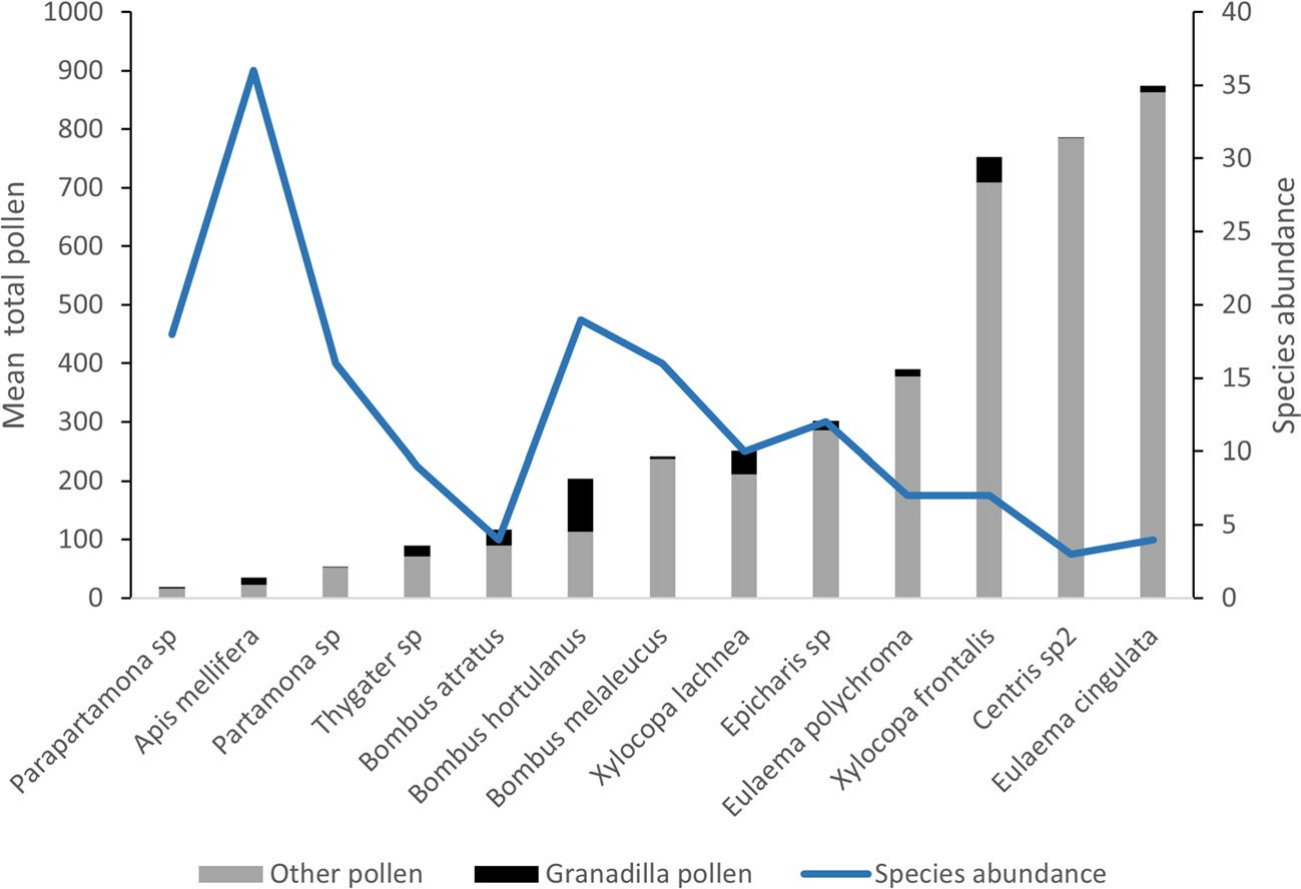
Mean total pollen grains for potential pollinator bees of the granadilla crop. We show the abundance of bees with more than three individuals in the community (blue line). Total pollen is represented by the two colors in a bar; the black represents granadilla pollen, and gray is the pollen from other plant species. The total pollen is the sum of black and gray in a bar

Since the insect species carrying the highest number of total pollen grains were species of the Apidae family, our analysis focused on this family (Table S2). Of the Apidae, the most representative species were *Eulaema cingulate* (Fabricius), with 873 mean total pollen carried (for more detail, see Table S2), followed by *Centris* sp., and *Xylocopa frontalis* (Olivier) carrying 786 and 752 mean total pollen grains (Fig. 1). The species with the highest number of granadilla pollen grains were *Bombus hortulanus* (Smith) (90.4), *X. frontalis* (43), *Xylocopa lachnea* (Moure) (41), *Bombus atratus* (Franklin) (27.7), and *Thygater* sp. (19.1) (Fig. 1, Table S2).

When analyzing the relationship between the abundance of bee species and the total pollen they can carry on their bodies, there is a negative strong relationship (*r* =  − 0.61), where the most abundant species such as *A. mellifera* carry less amounts of pollen.

### Functional traits and pollen carried by bees

**Fig. 2:**
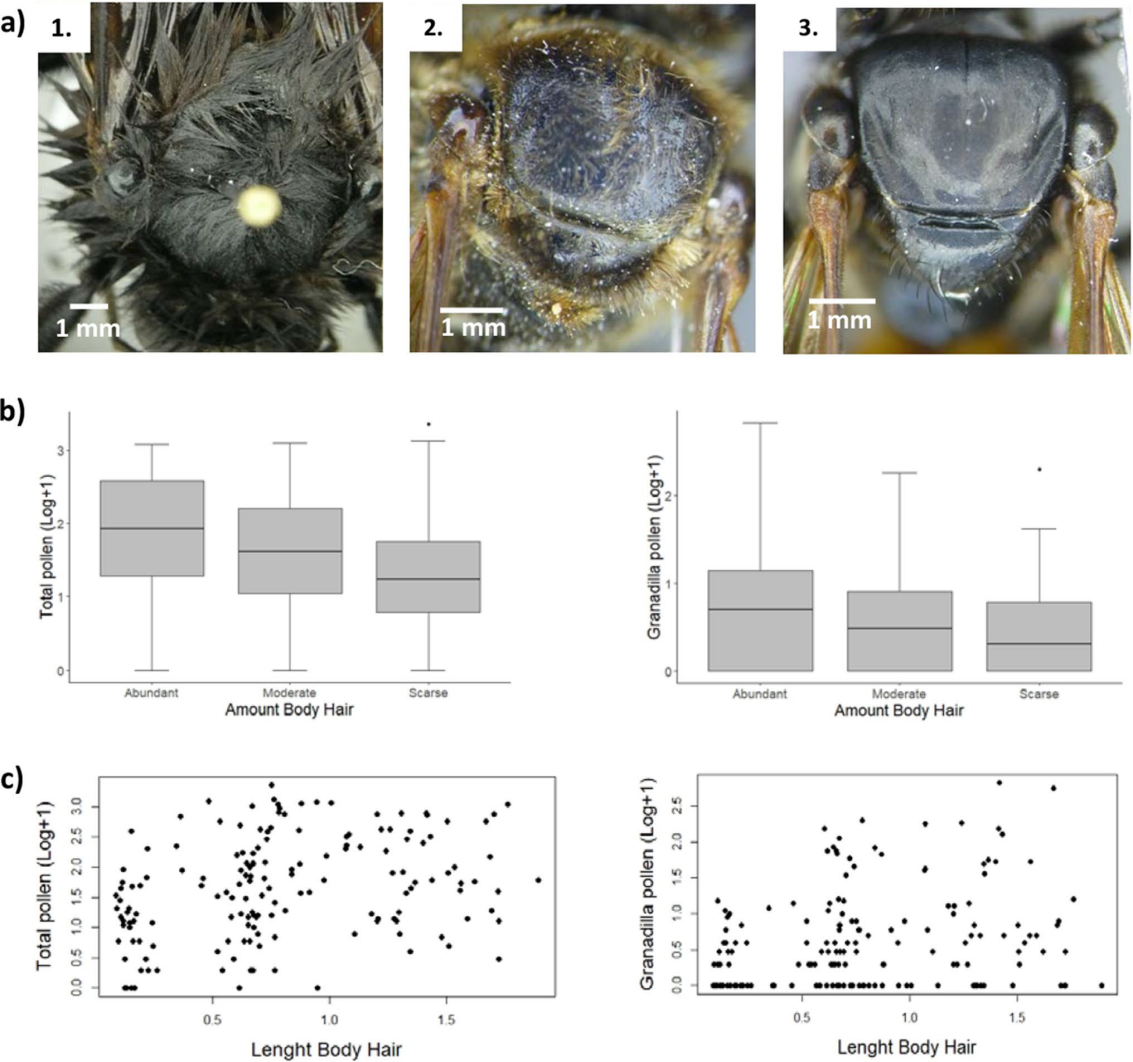
Amount and length of body hair of bees in granadilla crops. (**a**) Photographs of types of hairs found in the bees studied. (1) Long and abundant hairs, (2) Short and moderately abundant hairs, (3) Short and scarce hairs. (**b**) Comparison of the amount of body hair to the total of pollen samples compared only to samples with sweet granadilla pollen. (**c**) Relationship between analyzed body hair length and total pollen samples (left) and sweet granadilla pollen samples (right)

The bees found in the sweet granadilla flowers were characterized by three types of hairs: individuals with long and abundant hairs, individuals with short and moderate abundance hairs, and individuals with short and scarce hairs (Fig. 2a).

We found that the amount of body hair in bees favored the transport of a slightly greater quantity of total pollen (K-W = 13.5, *p* = 0.001; Fig. 2b). However, there were no statistical differences between the abundant, moderate, and scarce amount, when analyzing granadilla pollen (K-W = 4.19, *p* = 0.12). There was also no clear relationship between body hair length and the amount of total or sweet granadilla pollen that bees can carry (Fig. 2c).

Solitary bees transport more pollen than eusocial bees (total pollen: K-W = 55.8, *p* = 0.0001; granadilla pollen: K-W = 7.66, *p* = 0.02; see Fig. S3A and S3B), Likewise, underground nests and the scope structures to carry pollen are the traits that are more common when a greater amount of pollen is found on individuals (Fig. S3A and S3B).

**Fig. 3:**
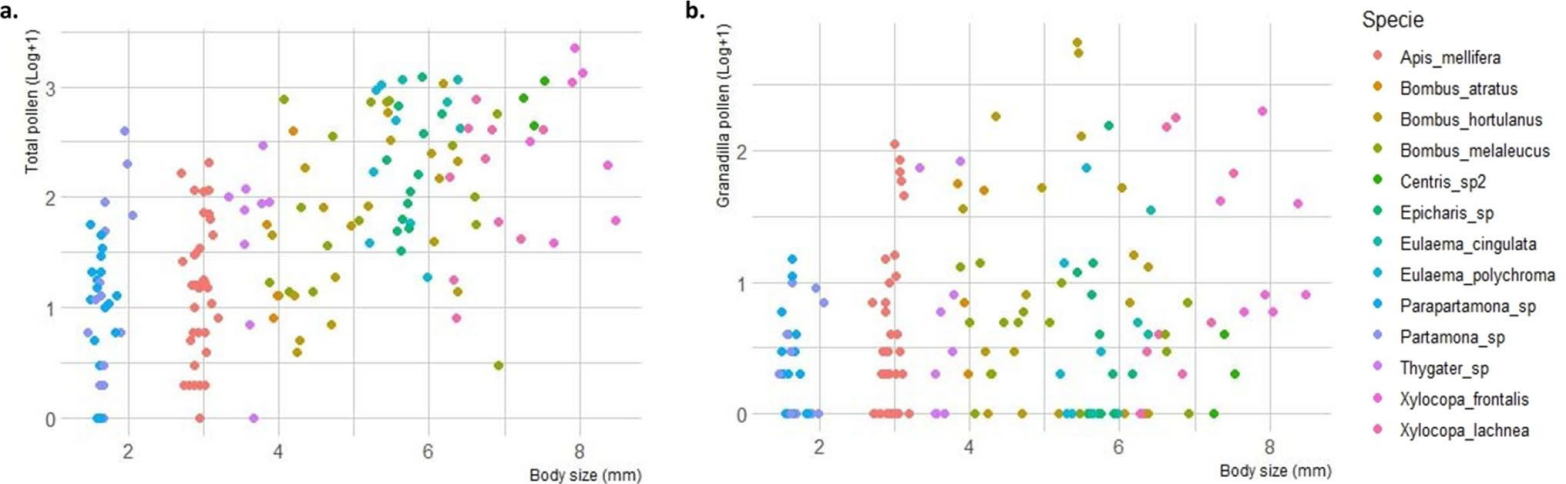
Body size and pollen (Log + 1) for individuals of each bee species. (**a**) Pollen total. (**b**) Sweet granadilla pollen

Body size did not show a pattern or have a marked relationship with pollen in general (Fig. 3a and 3b); however, there is a subtle tendency for large body sizes to transport more total pollen (Fig. 3a).

High collinearity was found between some traits. Length of the left wing and wingspan were eliminated from further analysis (Fig. S4). Regarding traits such as eating habits, we found that more than 90% of the individuals are nectarivores-polynivores. Therefore, for the subsequent analyses, the selected trait variables were as follows: body size, hair length, amount of body hair, structures to carry pollen, social behavior, and nest location.

### Cluster of traits

**Fig. 4:**
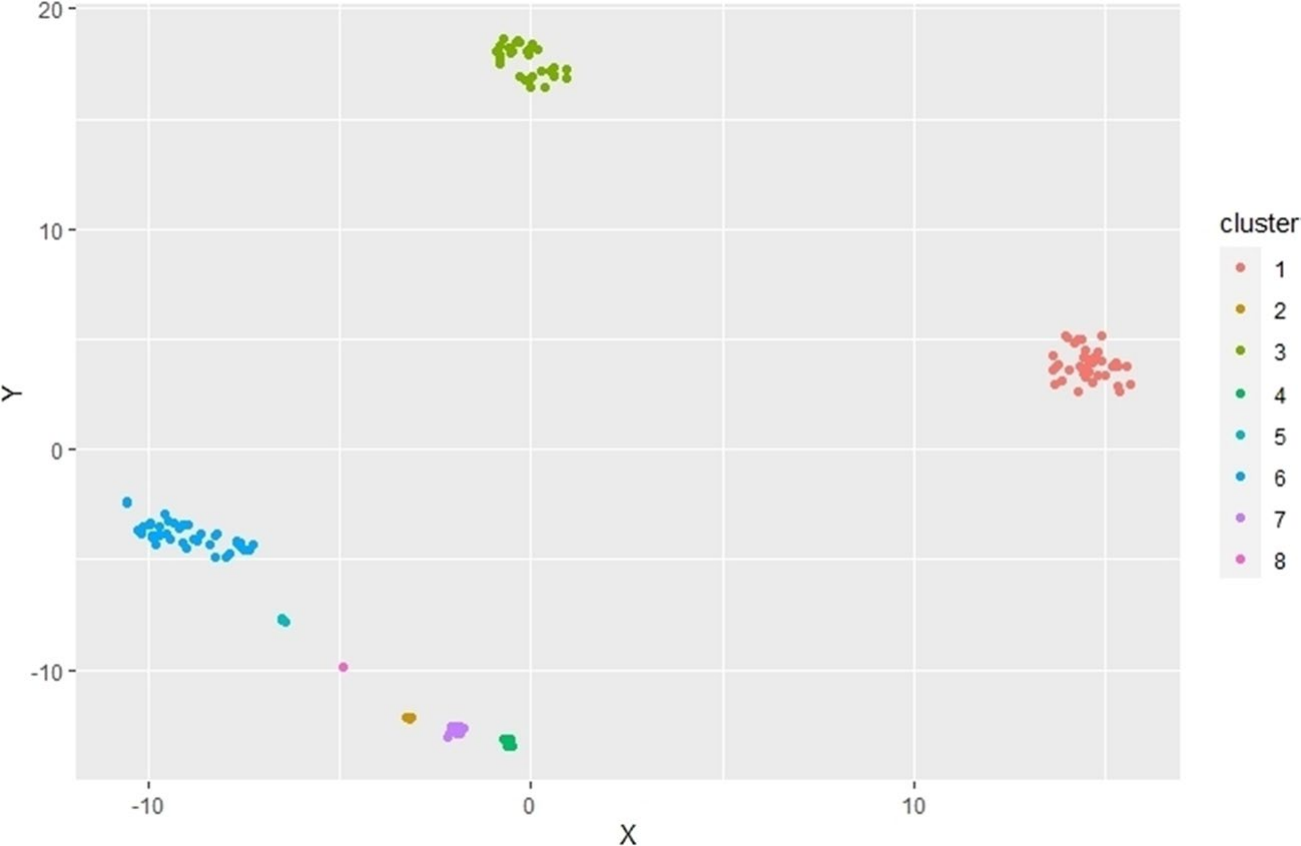
Grouping of individuals according to the functional traits evaluated. There are eight (8) groups differentiated by colors, and within each one of these the species we identified: Cluster: 1, *Apis mellifera*; 2, *Xylocopa frontalis*; 3, *Partamona* sp. and *Parapartamona* sp.; 4, *Epicharis* sp.; 5, *Eulaema cingulata* and *Eulaema. polychroma*; 6, *Bombus atratus*, *Bombus hortulanus* and *Bombus melaleucus*; 7, *Centris sp2* and *Xylocopa lachnea*; 8, *Thygater* sp

The cluster analysis showed that the data of the traits was grouped into 8 clusters (Fig. 4) and identifying each one (according to species) showed that not all the individuals were grouped according to species taxonomy. Some individuals whose traits are very characteristic compared to the others (small, eusocial, see Table 1) were in groups well-differentiated from the rest of the individuals, such as cluster 1 (*A. mellifera* group) and cluster 3 (*Partamona* sp. and *Parapartamona* sp. group, both belonging to the Meliponini tribe)*.* We saw groups whose traits were not taxonomically differentiated, as seen in the cluster 7, where we found individuals of the species *Centris sp2* and *X. lachnea* (Fig. 4).

**Table 1:** Functional trait values and standard deviations included in each cluster showed in Fig. 4. Nest location relative to the ground (above ground, underground)

Cluster	Species	Body size	Hair length	Social behavior	Amount body hair	Nest location	Structure carrying pollen	Granadilla pollen	Total pollen
		Mean (sd)	Mean (sd)					Mean (sd)	Mean (sd)
1	*Apis mellifera*	3.0 (0.10)	0.66 (0.052)	Eusocial	Moderate	Above	Corbícula	17.6 (29.9)	42.4 (55.7)
2	*Xylocopa frontalis*	8.0 (0.35)	0.96 (0.102)	Solitary	Scarse	Above	Corbícula	49.2 (74.8)	506.2 (560.9)
3	*Partamona* sp., *Parapartamona* sp.	1.6 (0.13)	0.15 (0.039)	Eusocial	Scarse	Above	Scopa	4.4 (3.8)	26.4 (19.5)
4	*Epicharis* sp.	5.8 (0.24)	0.52 (0.067)	Solitary	Abundant	Above	Corbícula	27.3 (56.5)	230.9 (226.1)
5	*Eulaema cingulata*, *Eulaema polychroma*	5.8 (0.52)	0.71 (0.136)	Solitary	Abundant	Above	Corbícula	18.7 (27.0)	442.6 (411.4)
6	*Bombus atratus*, *Bombus hortulanus*, *Bombus melaleucus*	4.9 (0.94)	1.4 (0.173)	Parasocial	Moderate	Under	Scopa	63.2 (154.9)	201.3 (277.8)
7	*Centris* sp., *Xylocopa lachea*	7.0 (0.45)	1.1 (0.256)	Solitary	Moderate	Above	Scopa	45.7 (71.4)	428.7 (348.0)
8	*Thygater*	3.7 (0.20)	0.75 (0.100)	Solitary	Abundant	Above	Scopa	28.7 (38.8)	102.2 (100.3)

### Relationship between traits and pollen

The minimum selected model only contained body size as a variable. This trait has a positive effect on total pollen and granadilla pollen. This positive trend exhibited less extent than in granadilla pollen (Table 2). The differences between the AIC values of the best-fitting model with the null model were greater than 2 in total pollen and in granadilla pollen. The selection of predictors by the best-fitting model for total and granadilla pollen agree with the results of the relative importance analysis (Table S3). When running the models with clusters as a variable, we found that none of these clusters explained pollen transport better than the selected body size model (Table 2).

**Table 2:** Influence of bee functional traits on total pollen and sweet granadilla pollen. The model estimates (and standard error) from the model with the lowest AICc are shown

	Model with total pollen as a response variable		Model with sweet granadilla pollen as a response variable	
	Model estimate (sd error)	AIC	Model estimate (sd error)	AIC
Intercept	2.20 (0.26)		1.34 (0.55)	
Body size	0.57 (0.05)		0.30 (0.09)	
AIC minimum adequate model		1238.0		864.6
AIC null model		1274.5		869.7
ΔAIC (null–minimum)		36.5		5.1
AIC cluster model		1256.4		876.6
Cluster 1	3.46 (0.29)		2.53 (0.40)	
Cluster 2	2.40 (0.52)		0.67 (0.64)	
Cluster 3	0.06 (0.45)		− 0.96 (0.50)	
Cluster 4	2.36 (0.57)		0.51 (0.62)	
Cluster 5	2.63 (0.50)		0.87 (0.62)	
Cluster 6	1.68 (0.31)		0.78 (0.43)	
Cluster 7	2.42 (0.43)		0.83 (0.56)	
Cluster 8	1.24 (0.53)		0.32 (0.62)	

## Discussion

### Sweet granadilla flower visitor and pollen transport

This study found that bees belonging to the Apidae family were the most frequent floral visitors to this crop that carried granadilla body pollen. *A. mellifera* was the most abundant species with almost half of the specimens collected. This is a non-native species widely distributed in Colombia, mainly due to beekeeping activities for honey production (ANDI 2017). Its use as a crop pollinator around the world is well-known (Aizen and Harder 2009; Garibaldi et al. 2017).

However, *A. mellifera* was the species with the lowest number of pollen grains (total or granadilla pollen) on its body and due to its small size in relation to all the sweet granadilla visiting the bee community. This specie may not be a species that efficiently pollinates the flower of this plant. *A. mellifera* is more like a pollen robber species (Junqueira et al. 2013) that possibly does not contribute to the pollination and production of sweet granadilla fruits and negatively affects the supply of pollen available with which native species carry out effective pollination and obtain food resources. This agrees with other studies of granadilla crops (Franco et al. 2007; Arias-Suarez et al. 2016). Studies had shown that *A. mellifera* and their dominance alter trophic interactions due to competition for the use of resources (Roubik 2009; Giannini et al. 2015; Garibaldi et al. 2021). Like *A. mellifera*, *Partamona* sp. and *Parapartamona* sp. have a great abundance, are small, and do not carry a high number of total pollen grains and can be classified like robber species in the granadilla crop.

Within the native species that visit the crop, *Xylocopa* and *Bombus* are the most species abundant genera and have been reported in various regions of the country as frequent flower-visitors of the sweet granadilla crop (Franco et al. 2007; Arias-Suarez et al. 2016; Gutiérrez-Chacón et al. 2018). We find that *B. hortulanus* is one of the species that gathered a higher number of granadilla pollen with its body, and together with its higher relative abundance suggested that it is one of the most frequent visitors and an effective pollinator of sweet granadilla in this part of the country.

On the other hand, we found species with lower abundances like *E. cigulata*, *Centris sp2*, and* X. frontalis* transported a greater amount of total pollen but a smaller amount of granadilla pollen. Although these species are not frequent visitors to the crop, they are efficient pollen transporters from various floral resources, near the granadilla crops.

### Functional traits of the community and relationships with the species

We found that the bee community in granadilla crops is mostly nectarivore-pollinivore and polylectic since they take pollen from different species of flowers (see Pollen morphotype, Table S2). This can bring them advantages such as improving the consumption of macronutrients (Vaudo et al. 2016) and reducing the presence of pathogens (Fowler et al. 2020).

When performing the cluster analysis by functional traits, we found that in general the groupings were formed following the traits. The first large group made up by *A. mellifera* and *Partamonas* and *Parapartamonas* group were mainly characterized as social species with small body sizes. A second group containing the rest of the bees are larger. Then we can see groupings that are very consistent with the taxonomy of the species, genera, or tribes. However, some species of bees were grouped differently. Species such as *X. lachnea* and *Centris* sp. can share traits with other species or tribes. These results show us that the traits can demonstrate the functional assembly of the potentially pollinating bee community in granadilla crops better than species compositions (Castro et al. 2020; Heino 2009).

### Interaction between functional traits and pollen transport

Analyzing the interaction between traits and body pollen loads, we found that there was no effect of hair length and the amount of body hair in transporting granadilla pollen or total pollen, although some studies have highlighted the importance of hairiness in pollen transport (Stavert et al. 2016; Roquer-Beni et al. 2020; Khan and Liu 2022). Body size is the trait that most influences the amount of granadilla and total pollen to be transported, suggesting that the larger bees can transport more pollen, in this case, the pollen is transported areas such as the thorax and abdomen. This is consistent with several studies that show a positive relationship between the body size of individuals and the number of pollen grains they can carry on their bodies (Garibaldi et al. 2015; Cullen et al. 2021; Földesi et al. 2021). In studies of granadilla plants, whose pollinators are large bee species, the bees increase fruit formation (70%), fruit weight, and fruit seed, generally improving granadilla production (Arias-Suarez et al. 2016; Gutiérrez-Chacón et al. 2018).

Another important aspect evident in different studies is that *Xylocopa* bees are the main pollinating agent of granadilla. However, we found that *B. hortulanus* was the main pollinator, and *X. frontalis* was less abundant than *X. lachnea*, showing that the body size traits are a better predictor of granadilla pollination than species composition. However, it would be interesting to understand the effects of environmental variables and landscape configuration in determining the presence and abundance of these larger bees in this fruit crop, so as to increase the transporting of granadilla pollen and enhance granadilla pollination.

Likewise, it was clear that when trying to see if these traits were influenced by the species taxonomy or if they showed interactions, through the grouping of the traits in functional groups, none of the models was better in total pollen transport of granadilla compared to the model with only body size as a predictor variable. And although traits of sweet granadilla flowers were not measured in this study, we can say that they are large and their stigma and anthers protrude from the corolla, which makes it necessary for large bees to visit them so that they can have contact with the flowers anthers and stigma of the flowers (Arias-Suarez et al. 2016), as studies of other passionflower species have shown (Koschnitzke and Sazima 1997; Ángel-Coca et al. 2011).

All of this clearly shows that body size is a trait with great importance when evaluating the function of pollination in granadilla crops and other plants. Likewise, it is an easy trait to measure and is not expensive, compared with the measurement of hairiness, which is time-consuming and requires sophisticated equipment (Stavert et al. 2016; Roquer-Beni et al. 2020; Khan and Liu 2022). However, we suggest that the measurement traits from flowers, not evaluated in this study, like characteristic pollen morphology (diversity of shapes and structures of the exine, and the outer cuticularized wall of a pollen grain) possibly contributes to adherence to a bee pollinator, regardless of the amount or size of the hairs. Further research on the morphological characteristics of the pollen and its relationship to adherence to insects is necessary. Likewise, it is of great importance to evaluate other types of traits that intervene in pollination, not only of the insect, but also of the flowers of the plants that are being pollinated.

The length of the hairs was not very important in the transport of pollen of sweet granadilla; however, large-bodied bees are the ones that transport more granadilla pollen and are good pollinators of this fruit crop. When evaluating what traits are important for pollination, it is important to consider the morphological traits of the flowers that bees visit that can show us the compatibility between insects and flowers and can indicate which traits are really important in pollination. For example, pollen morphology possibly helps the pollen to adhere to individual bees that have short or long hairs. However, more research is required from a trait-matching perspective (Garibaldi et al. 2015; Brousseau et al. 2018) in which other traits must be analyzed at the level of bees, as well as at the level of plants, like granadilla flowers and pollen and their morphological characteristics.

To favor and maintain crop pollination and obtain greater production, a knowledge of small farmers about the importance of pollinators and of carrying out conservation actions in their territory must be reinforced, following some guidelines that already exist (e.g., Colombian pollinator initiative, Nates-Parra 2016).

## Supplementary Information

Below is the link to the electronic supplementary material.

Supplementary file1 (DOCX 1305 KB)

## Data Availability

All the data associated with the manuscript are part of the attached supplementary material.

## References

[CR1] Aizen MA, Harder LD (2009). The global stock of domesticated honey bees is growing slower than agricultural demand for pollination. Curr Biol.

[CR2] ANDI (2017). Línea base de la población de apicultores en Colombia. Cámara de procultivos de la ANDI. https://abejasenagricultura.org/wp-content/uploads/2018/07/Linea-de-Base-Poblacion-Apicultores.pdf. Accessed 4 November 2021

[CR3] Ángel-Coca C, Nates-Parra G, Ospina-Torres R, Melo Ortiz CD, Amaya-Márquez M (2011). Floral and reproductive biology of the“ gulupa” Passiflora edulis Sims f. edulis. Caldasia.

[CR4] Arias-Suarez JC, Ocampo-Pérez J, Urrea-Gómez R (2016). Sistemas de polinización en granadilla (Passiflora ligularis Juss) como base para estudios genéticos y de conservación. Acta Agronómica.

[CR5] Bartón K (2020). MuMIn: multi-model inference. R Package Version.

[CR6] Bates D, Mächler M, Bolker B, Walker S (2015). Fitting linear mixed-effects models using lme4. Journal of Statistical Software.

[CR7] Borges RC, Padovani K, Imperatriz-Fonseca VL, Giannini TC (2020). A dataset of multi-functional ecological traits of Brazilian bees. Scientific Data.

[CR8] Brousseau PM, Gravel D, Handa IT (2018). On the development of a predictive functional trait approach for studying terrestrial arthropods. J Animal Ecol.

[CR9] Castro FSD, Da Silva PG, Solar R, Fernandes GW, Neves FDS (2020). Environmental drivers of taxonomic andfunctional diversity of ant communities in a tropical mountain. Insect Conserv Divers.

[CR10] Crenna E, Sala S, Polce C, Collina E (2017). Pollinators in life cycle assessment: towards a framework for impact assessment. J Clean Prod.

[CR11] Cullen N, Xia J, Wei N, Kaczorowski R, Arceo-Gómez G, O’Neill E, Hayes R, Ashman TL (2021). Diversity and composition of pollen loads carried by pollinators are primarily driven by insect traits, not floral community characteristics. Oecologia.

[CR12] da Encarnacao Coutinho JG, Garibaldi LA, Viana BF (2018). The influence of local and landscape scale on single response traits in bees: a meta-analysis. Agr Ecosyst Environ.

[CR13] Földesi R, Howlett BG, Grass I, Batáry P (2021). Larger pollinators deposit more pollen on stigmas across multiple plant species-a meta-analysis. J Appl Ecol.

[CR14] Fornoff F, Kein AM, Hartig F, Benadi G, Venjakob C, Schaefer HM, Ebeling A (2017). Functional flower trait and their diversity drive pollinator visitation. Oikos.

[CR15] Fowler AE, Stone EC, Irwin RE, Adler LS (2020). Sunflower pollen reduces a gut pathogen in worker and queen but not male bumble bees. Ecol Ent.

[CR16] Fox J, Weisberg S (2019). An R companion to applied regression, Third edition. Sage, Thousand Oaks CA. https://socialsciences.mcmaster.ca/jfox/Books/Companion/

[CR17] Franco Y, Alzate F, Peláez JM (2007). Factores ambientales incidentes en la población de Xylocopa y su efecto en el cultivo de granadilla en tres veredas del municipio de Guarne (Colombia). Rev Univ Católica Orien.

[CR18] Gallai N, Salles JM, Settele J, Vaissière BE (2009). Economic valuation of the vulnerability of world agriculture confronted with pollinator decline. Ecol Econ.

[CR19] Garibaldi LA, Steffan-Dewenter I, Kremen C, Morales JM, Bommarco R, Cunningham S, Calvalheiro LG, Chacoff NP, Dudenhöffer JH, Greendeafm SS, Holzschuh A, Isaacs R, Krewenka K, Mandelik Y, Mayfield MM, Moradin LA, Potts SG, Ricketts TH, Szentgyögyi H (2011). Stability of pollination services decreases with isolation from natural areas despite honey bee visits. Ecol Lett.

[CR20] Garibaldi LA, Bartomeus I, Bommarco R, Klein AM, Cunningham SA, Aizen MA, Boreux V, Garratt MPD, Carvalheiro LG, Kremen C, Morales CL, Schüepp C, Chacoff NP, Freitas BM, Gagic V, Holzschuh A, Klatt BK, Krewenka KM, Krishnan S (2015). Trait matching of flower visitors and crops predicts fruit set better than trait diversity. J Appl Ecol.

[CR21] Garibaldi LA, Requier F, Rollin O, Andersson GKS (2017). Towards an integrated species and habitat management of crop pollination. Current Opinion in Insect Science.

[CR22] Garibaldi LA, Pérez-Méndez N, Cordeiro GD, Hughes A, Orr M, Alves-dos-Santos I, Freitas BM, Freitas de Oliveira F, LeBuhn G, Bartomeus I, Aizen MA (2021). Negative impacts of dominance on bee communities: does the influence of invasive honey bees differ from native bees?. Ecology.

[CR23] Giannini TC, Garibaldi LA, Acosta AL, Silva JS, Maia KP, Saraiva AM, Guimarães PR, Kleinert AMP (2015). Native and non-native supergeneralist bee species have different effects on plant-bee networks. PLoS ONE.

[CR24] Goulnik J, Plantureux S, Van Reeth C, Baude M, Mesbahi G, Michelot-Antalik A (2020). Facial area and hairiness of pollinators visiting semi-natural grassland wild plants predict their facial pollen load. Ecological Entomology.

[CR25] Gutiérrez-Chacón C, Fornoff F, Ospina-Torres R, Klein AM (2018). Pollination of granadilla (*Passiflora ligularis*) benefits from large wild insects. J Econ Entomol.

[CR26] Heino J (2009). Biodiversity of aquatic insects: spatial gradients and environmental correlates of assemblage-level measures at large scales. Freshwater Rev.

[CR27] Hernández L, Castillo F, Ocampo J, Wyckhuys KA (2011). Guía de identificación de plagas y enfermedades para la Maracuyá, la Gulupa y la Granadilla.

[CR28] Howlett BG, Walker MK, Rader R, Butler RC, Newstrom-Lloyd LE, Teulon DAJ (2011). Can insect body pollen counts be used to estimate pollen deposition on pak choi stigmas. New Zealand Plant Protection.

[CR29] Junqueira CN, Yamamoto M, Oliveira PE, Hogendoorn K, Augusto SC (2013). Nest management increases pollinator density in passion fruit orchards. Apidologie.

[CR30] Kaufman L, Rousseeuw PJ (2005). Finding groups in data: an introduction to cluster analysis (Wiley series in probability and statistics).

[CR31] Khan KA, Liu T (2022). Morphological structure and distribution of hairiness on different body parts of *Apis mellifera* with an implication on pollination biology and a novel method to measure the hair length. Insects.

[CR32] Klein A-M, Vaissiére BE, Cane JH, Steffan-Dewenter I, Cunningham SA, Kremen C, Tscharntke T (2007). Importance of pollinators in changing landscapes for world crops. Proc Biol Sci.

[CR33] Koschnitzke C, Sazima M (1997). Biologia floral de cinco espécies de Passiflora L. (Passifloraceae) em mata semidecídua. Brazilian J Botany.

[CR34] Martín-López B, González JA, Díaz SI, Castro I, García-Llorente M (2007). Biodiversidad y bienestar humano: el papel de la diversidad funcional. Ecosistemas.

[CR35] Mayfield MM, Ackerly D, Daily GC (2006). The diversity and conservation of plant reproductive and dispersal functional traits in human-dominated tropical landscapes. J Ecol.

[CR36] Melgarejo LM (2015). Granadilla (*Passiflora ligularis* Juss): Caracterización Ecofisiológica del cultivo. Universidad Nacional de Colombia (Sede Bogotá); Colciencias; Corporación Centro de desarrollo tecnológico de las Passifloras de Colombia- CEPASS. Bogotá, Colombia. ISBN: 978–958–775–396–7

[CR37] Moretti M, De Bello F, Roberts SP, Potts SG (2009). Taxonomical vs. functional responses of bee communities to fire in two contrasting climatic regions. J Anim Ecol.

[CR38] Munyuli T (2014). Influence of functional traits on foraging behavior and pollination efficiency of wild social and solitary bees visiting coffee (*Coffea canephora*) flowers in Uganda. Grana.

[CR39] Nates-Parra G (2016). Iniciativa Colombiana de Polinizadores - Abejas – ICIPA.

[CR40] Ocampo Pérez J (2007). Diversidad de las Passifloraceae colombianas: biogeografía y una lista actualizada para la conservación. Biota Colombiana.

[CR41] Parker AJ, Tran JL, Ison JL, Bai JDK, Weis AE, Thomson JD (2015). Pollen packing affects the function of pollen on corbiculate bees but not non-corbiculate bees. Arthropod-Plant Interactions.

[CR42] Parra M. (2013). Acuerdo de competitividad para la cadena productiva de pasifloras en Colombia. Asohofrucol, Cepass, Consejo Nacional de Pasifloras, Ministerio de Agricultura y Desarrollo Rural, Bogotá-Colombia.

[CR43] PDT-Algeciras, 2016–2019. Plan de desarrollo territorial del municipio de Algeciras, Huila. http://www.algeciras-huila.gov.co/planes/plan-de-desarrollo-20162019. Accessed 15 June 2021

[CR44] Potts SG, Biesmeijer JC, Kremen C, Neumann P, Schweiger O, Kunin WE (2010). Global pollinator declines: trends, impacts and drivers. Trends Ecol Evol.

[CR45] R Development Core Team (2020). R: a language and environment for statistical computing. R Foundation for Statistical Computing, Vienna, Austria. http://www.R-project.org

[CR46] Roquer-Beni L, Rodrigo A, Arnan X, Klein AM, Fornoff F, Boreux V, Bosch J (2020). A novel method to measure hairiness in bees and other insect pollinators. Ecol Evol.

[CR47] Roubik DW (2009). Ecological impact on native bees by the invasive Africanized honey bee. Acta Biológica Colombiana.

[CR48] Roubik DW (Ed.). (1995). Pollination of cultivated plants in the tropics. Agricultural Services Bulletin 118. Food and Agriculture Organization of the United Nations.

[CR49] Schneider CA, Rasband WS, Eliceiri KW (2012). NIH Image to ImageJ 25 years of image analysis. Nature Methods.

[CR50] Shipley B, De Bello F, Cornelissen JHC, Laliberté E, Laughlin DC, Reich PB (2016). Reinforcing loose foundation stones in trait-based plant ecology. Oecologia.

[CR51] Stavert JR, Liñán-Cembrano G, Beggs JR, Howlett BG, Pattemore DE, Bartomeus I (2016). Hairiness: the missing link between pollinators and pollination. PeerJ.

[CR52] Vaudo AD, Patch HM, Mortensen DA, Tooker JF, Grozinger CM (2016). Macronutrient ratios in pollen shape bumble bee (*Bombus impatiens*) foraging strategies and floral preferences. PNAS.

[CR53] Wickham H, Averick M, Bryan J, Chang W, McGowan LD, François R, Grolemund G, Hayes A, Henry L, Hester J, Kuhn M, Pedersen TL, Miller E, Bache SM, Müller K, Ooms J, Robinson D, Seidel BD, Spinu V (2019). Welcome to the tidyverse. J Open-Source Software.

[CR54] Woodcock BA, Garratt MPD, Powney GD, Shaw RF, Osborne JL, Soroka J, Lindström SAM, Stanley D, Ouvrard P, Edwards ME, Jauker F, McCracken MM, Zou Y, Potts SG, Rundlöf M, Noriega JA, Greenop A, Smith HG, Bommarco R (2019). Meta-analysis reveals that pollinator functional diversity and abundance enhance crop pollination and yield. Nat Commun.

