# Supplementary material for: Functional Traits in Bees: the Role of Body Size and Hairs in the Pollination of a Passiflora Crop
Source: Neotrop Entomol. 2023 Jul 26;52(4):642–51. doi: 10.1007/s13744-023-01058-w (PMC10390375; doi:10.1007/s13744-023-01058-w)
Supplement: Supplementary file 1 — Supplementary file1 (DOCX 1305 KB) [file 13744_2023_1058_MOESM1_ESM.docx]

**Supplementary Materials**

**“Functional traits in bees: the role of body size and hairs in the pollination of a Passiflora crop”**

Journal: **Neotropical Entomology**

**Angela M. Cortés-Gómez^1*^;** **Adrián González-Chaves ^2^; Nicolás Urbina-Cardona^3^; Lucas A. Garibaldi^4,5^**

^1^Facultad de Estudios Ambientales y Rurales. Pontificia Universidad Javeriana, Bogotá, Colombia. ORCID ID 0000-0002-1283-9810.

^2^Departamento de Ecología, Instituto de Biociências. Universidade de São Paulo, Brasil. ORCID ID 0000-0002-5233-8957

^3^Facultad de Estudios Ambientales y Rurales. Pontificia Universidad Javeriana, Bogotá, Colombia. ORCID ID 0000-0002-4174-8467

^4^Universidad Nacional de Rio Negro, Bariloche, Instituto de Investigaciones en Recursos Naturales, Agroecología y Desarrollo Rural, Río Negro, Argentina. ORCID ID 0000-0003-0725-4049

^5^Consejo Nacional de Investigaciones Científicas y Técnicas, Instituto de Investigaciones en Recursos Naturales, Agroecología y Desarrollo Rural, Río Negro, Argentina.

**^*^Corresponding author AMC-G: cortesangela@javeriana.edu.co**

**Supplementary Materials**

**Content**

Figures: Fig. S1 - S2 - S3 – S4

Tables: Table S1 – S2 – S3

**Figures**

**A.**


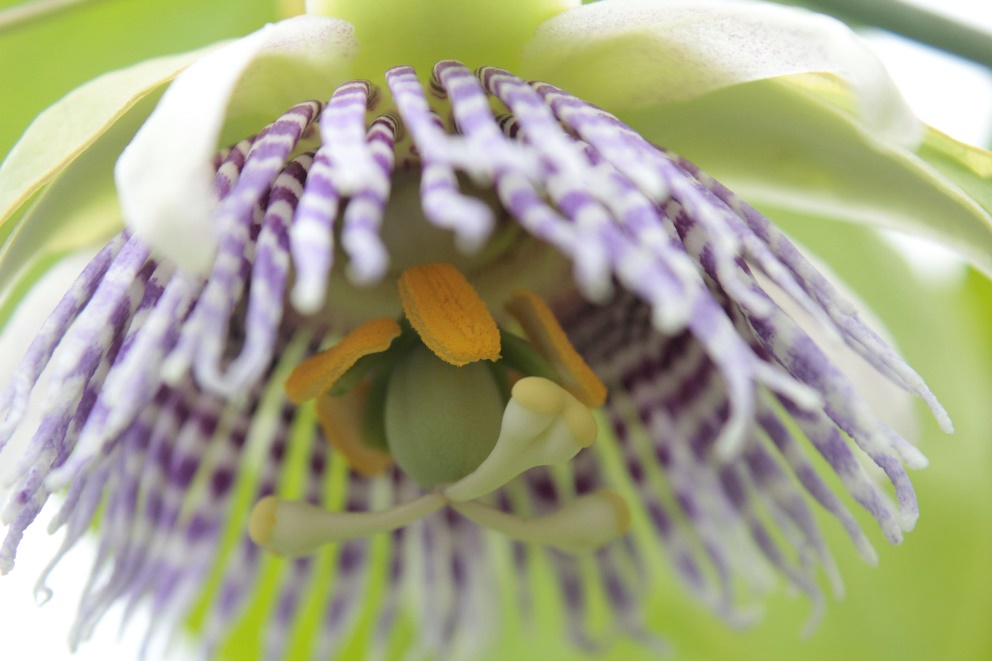


**B.**

**
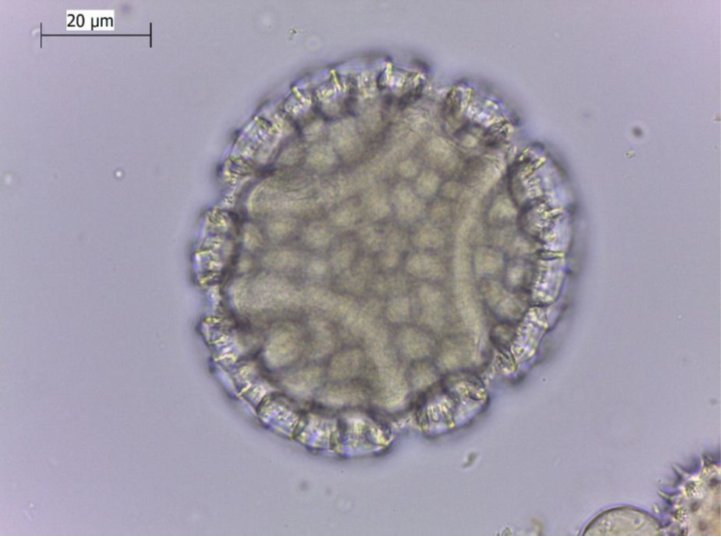
**

**Fig S1**. A. Photo of a granadilla flower with its anthers loaded with pollen (Author: Angela Cortés-Gómez). B. Photo of a Sweet granadilla pollen grain 100x. (Author: Susana Currea).


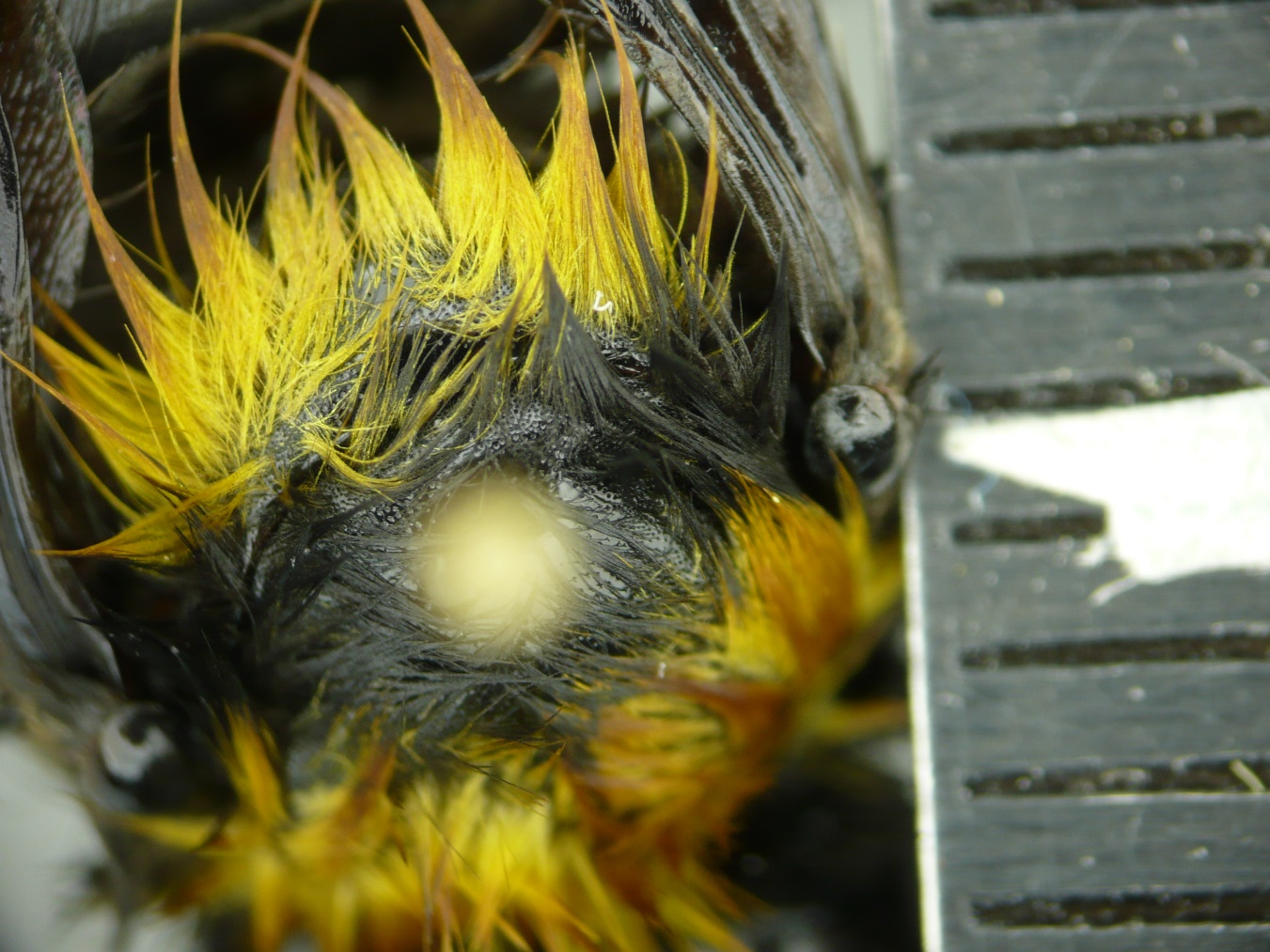


**2**

**3**

**1**

**Fig S2**. Photo of the thorax of a bee showing an example of how the measurements of the length of the hairs were taken. For each bee, measurements of the hair length were taken in three parts of the thorax. The measurements were taken from the base to the tip of the hair.


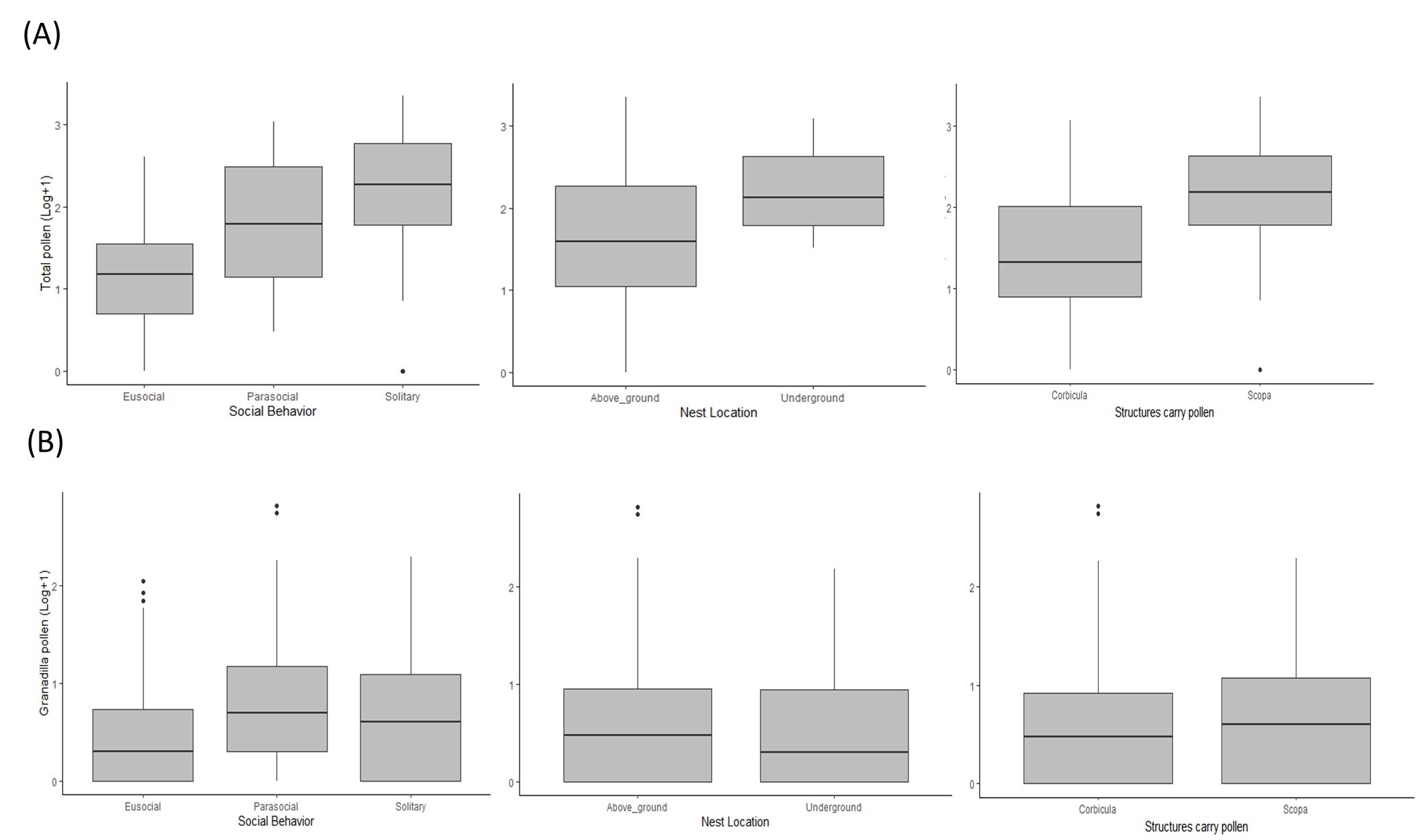


**Fig. S3**. Box plot of the amount of total pollen (A) and sweet granadilla pollen (B). Dark bar is the median, width of bar represents the 25th and 75th percentiles; the whiskers are the minimum and maximum excluding outliers.


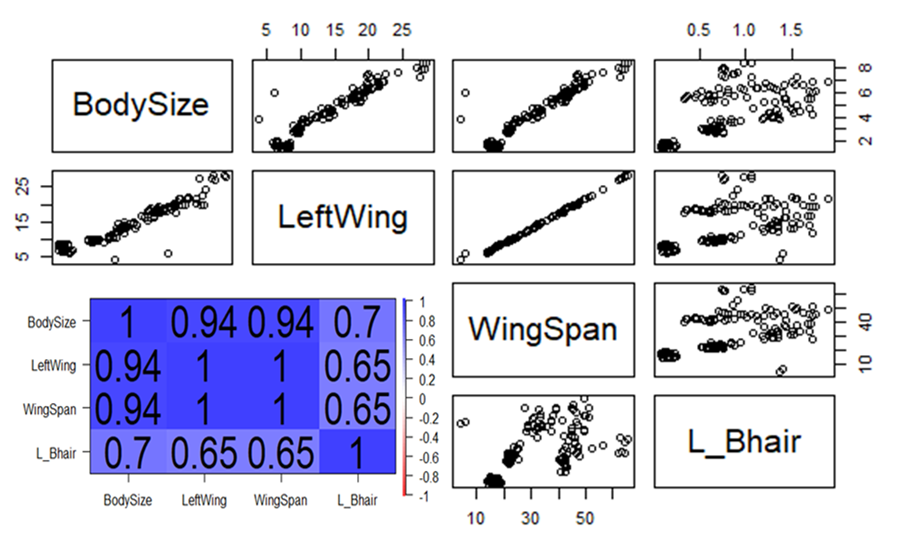


**Fig. S4**. Spearman's correlation between the morphometric traits of bees found in sweet granadilla crops.

**Tables**

**Table S1.** Functional traits selected for bees, that help to make the pollination function.

**Table S2.** Species abundance, functional traits and pollen count in samples of insect in sweet granadilla flowers.

**Table S3.** Influence of bee traits on transport of total pollen and granadilla pollen. The relative importance of each variable based on Akaike model weights is shown.

**REFERENCES**

Cane J H (1987). Estimation of bee size using intertegular span (Apoidea). Journal of the Kansas Entomological Society, 145-147.

Cane JH, Sipes S (2006). Floral specialization by bees: analytical methods and a

revised lexicon for oligolecty. In: Waser, N.M., Ollerton, J. (Eds.), Plant-Pollinator

Interactions: From Specialization to Generalization. University of Chicago Press,

Chicago, IL, pp. 99–122.

Carrié R, Andrieu E, Cunningham, SA, Lentini PE, Loreau M, Ouin A (2017). Relationships among ecological traits of wild bee communities along gradients of habitat amount and fragmentation. Ecography 40(1):85-97. https://doi.org/10.1111/ecog.02632

Crespi BJ, Yanega D (1995). The definition of eusociality.  Behavioral Ecology 6(1):109–115. https://doi.org[/10.1093/beheco/6.1.109](https://doi.org/10.1093/beheco/6.1.109)

Geslin B, Oddie M, Folschweiller M, Legras G, Seymour CL, Van Veen F F, Thébault E (2016). Spatiotemporal changes in flying insect abundance and their functional diversity as a function of distance to natural habitats in a mass flowering crop. Agriculture, Ecosystems & Environment 229: 21-29.

Moretti M, Dias AT, De Bello F, Altermatt F, Chown SL, Azcarate FM, Bell JR, Fournier B, Hedde M, Hortal J, Ibanez S, Öckinger De, Sousa JP, Ellers J, Berg MPM (2017). Handbook of protocols for standardized measurement of terrestrial invertebrate functional traits. Functional Ecology 31(3):558-567. https://doi.org/10.1111/1365-2435.12776

Michener CD (2000). *The bees of the world*. The Johns Hopkins University Press.

Parker AJ, Tran JL, Ison JL, Bai JDK, Weis AE, Thomson JD (2015). Pollen packing affects the function of pollen on corbiculate bees but not non-corbiculate bees. Arthropod-Plant Interactions 9(2):197-203. https://doi.org/10.1007/s11829-015-9358-z

Sohlström EH, Marian L, Barnes AD, Haneda NF, Scheu S, Rall BC, Brose U, Jochum M (2018). Applying generalized allometric regressions to predict live body mass of tropical and temperate arthropods. Ecology and evolution, 8(24):12737-12749.
